# Overexpression of the protein disulfide isomerase AtCYO1 in chloroplasts slows dark-induced senescence in *Arabidopsis*

**DOI:** 10.1186/s12870-018-1294-5

**Published:** 2018-05-04

**Authors:** Jun Tominaga, Yasutoshi Nakahara, Daisuke Horikawa, Ayumi Tanaka, Maki Kondo, Yasuhiro Kamei, Tsuneaki Takami, Wataru Sakamoto, Kazutoshi Unno, Atsushi Sakamoto, Hiroshi Shimada

**Affiliations:** 1 Department of Mathematical and Life Sciences, Graduate School of Science, Hiroshima University, 1-3-1 Kagamiyama, Higashi-Hiroshima, 739-8526 Japan; 2 Institute of Low Temperature Science, Hokkaido University, N19 W8, Kita-ku, Sapporo, 060-0819 Japan; 3 National Institute for Basic Biology, Okazaki, Aichi 444-8585 Japan; 4 Institute of Plant Science and Resources, Okayama University, Kurashiki, Okayama, 710-0046 Japan; 5 Laboratory of Electron Microscopy, University Hospital, Mizonokuchi, Teikyo University School of Medicine, 5-1-1, Futako, Takatsu-ku, Kawasaki, Kanagawa 213-8507 Japan

**Keywords:** DnaJ-like zinc-finger protein, Stay-green, Redox, Proteolysis, Ectopic expression, *Arabidopsis thaliana*

## Abstract

**Background:**

Chlorophyll breakdown is the most obvious sign of leaf senescence. The chlorophyll catabolism pathway and the associated proteins/genes have been identified in considerable detail by genetic approaches combined with stay-green phenotyping. *Arabidopsis* CYO1 (AtCYO1), a protein disulfide reductase/isomerase localized in the thylakoid membrane, is hypothesized to assemble the photosystem by interacting with cysteine residues of the subunits.

**Results:**

In this study, we report that ectopic overexpression of *AtCYO1* in leaves induces a stay-green phenotype during darkness, where oxidative conditions favor catabolism. In *AtCYO1ox* leaves, Fv/Fm and both chlorophyll *a* and chlorophyll *b* content remained high during dark-induced senescence. The thylakoid ultrastructure was preserved for a longer time in *AtCYO1ox* leaves than in wild type leaves. *AtCYO1ox* leaves maintained thylakoid chlorophyll-binding proteins associated with both PSII (D1, D2, CP43, CP47, LHCB2, and Cyt *f*) and PSI (PSA-A/B), as well as stromal proteins (Rubisco and ferredoxin-NADP+ reductase). *AtCYO1ox* did not affect senescence-inducible gene expression for chlorophyll catabolism or accumulation of chlorophyll catabolites.

**Conclusions:**

Our results suggest that ectopic overexpression of *AtCYO1* had a negative impact on the initiation of chlorophyll degradation and proteolysis within chloroplasts. Our findings cast new light on the redox regulation of protein disulfide bonds for the maintenance of functional chloroplasts.

**Electronic supplementary material:**

The online version of this article (10.1186/s12870-018-1294-5) contains supplementary material, which is available to authorized users.

## Background

In chloroplasts, chlorophyll is essential for harvesting light and transferring its excitation energy to the electron transport chain. To be functional, chlorophyll must be harnessed to chlorophyll-binding proteins in the thylakoid membrane, forming photosystems I and II (PSI and PSII) [1]. The photosystems consist of a core complex and a peripheral light-harvesting complex (LHC). In green plants, the core proteins bind only chlorophyll *a*, whereas the LHC protein (LHCP) binds both chlorophyll *a* and chlorophyll *b*. In oxygenic photosynthesis, free chlorophyll and its intermediate derivatives inevitably react with oxygen molecules as a photosensitizer, generating toxic singlet oxygen radicals [1]. Consequently, biogenesis and degradation of the photosynthetic apparatus within chloroplasts need to be tightly coordinated with those of chlorophyll. In principle, chlorophyll-depleted apoproteins never exist alone, whereas free chlorophyll immediately induces bleaching [2]. In the disassembly of the thylakoid photosystem, magnesium dechelation of chlorophyll *a* catalyzed by STAY-GREEN (SGR) is a crucial step for degradation of chlorophyll-protein complexes [2], whereas chlorophyll *b* reductase (CBR) initiates degradation of LHCP in PSII (LHCB) [3, 4]. Loss-of-function mutants in SGR or CBR delay chlorophyll degradation during senescence and show stay-green phenotypes [2, 4].

In *Arabidopsis*, CYO1 (AtCYO1)/SCO2 is a cotyledon-specific chloroplast biogenesis factor whose knockout mutants, *atcyo1*/*sco2*, develop albino/pale cotyledons [5, 6]. Despite being lethal to cotyledons, these mutants develop chloroplasts normally in rosette leaves, ultimately achieving growth and development that is comparable to wild type (WT). In darkness, *atcyo1* mutants develop etioplasts as do WT plants, indicating that AtCYO1 functions during photomorphogenesis [7]. AtCYO1 is tightly embedded in the thylakoid and co-exists with both PSI-LHCI and PSII-LHCII supercomplexes [5]. Like *Escherichia coli* DnaJ, AtCYO1 has a C_4_-type zinc-finger domain that catalyzes reduction of cysteine (Cys) thiols or isomerization of protein disulfide bonds, leading to the hypothesis that AtCYO1 acts as a molecular chaperone in the construction of the thylakoid photosystem [5]. Based on high amino acid sequence similarity, CYO1 orthologs were recently confirmed in rice (OsCYO1 [8]) and *Lotus japonicas* (LjSCO2 [9]). Intriguingly, mutants in these genes resulted in distinct cotyledon and leaf phenotypes, indicating that their physiological roles are not limited to cotyledons.

The de novo assembly and repair of PSII involve many common steps that are mediated by more than 40 proteins expressed stably or transiently [10]. THYLAKOID FORMATION1/NON-YELLOW COLORING4 (THF1/NYC4), the factor required for organizing mature thylakoids [11], was suggested to regulate the dynamics of PSII-LHCII supercomplexes during high-light stress [12, 13]. Like AtCYO1/SCO2 [14], THF1 interacts with LHCB [13] and is hypothesized to mediate the transport of LHCB in vesicles [15]. However, investigation of the potentially divergent roles of AtCYO1 has been limited, owing to the lethality of *atcyo1* to chloroplasts upon illumination. We thus investigated the effect of AtCYO1 on senescence, in which THF1/NYC4 has been shown to affect the rate of chlorophyll degradation [13, 16]. *Arabidopsis* rosette leaves were modified to ectopically overexpress AtCYO1 and subjected to dark incubation to stimulate senescence. We demonstrate how AtCYO1 impacts the stability of the photosynthetic apparatus.

## Results

### Overexpressed AtCYO1 appears in chloroplasts of rosette leaves

**Fig. 1:**
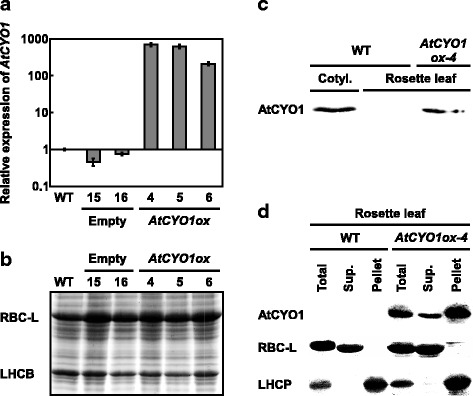
Comparison of mRNA and protein expression in wild-type (WT) and transgenic plants. Empty, empty vector; *AtCYO1ox*, *AtCYO1* overexpression vector. **a** Expression of *AtCYO1* in transgenic lines relative to WT (mean ± SE; *n* = 3). **b** SDS-PAGE gel with Coomassie brilliant blue staining of photosynthetic proteins in crude extracts. RBC-L, large subunit of Rubisco; LHCP, light-harvesting complex protein. **c** Accumulation of AtCYO1 protein in cotyledons and rosette leaves. **d** Accumulation of AtCYO1 in pelleted chloroplasts and supernatants (Sup.) of rosette leaves. In **b**, **c** and **d**, the same amount of sample was loaded in all lanes (400 μg fresh weight in **b**; 50 μg protein in **c**, **d**). In **a**, **b** and **d**, samples were taken from 2-week-old plants. In **c**, samples were taken from 7-day-old seedlings for cotyledons and 4-week-old plants for rosette leaves

We previously showed that *AtCYO1* in WT plants is almost exclusively expressed in cotyledons [5]. *AtCYO1* overexpression lines (*AtCYO1ox*) had mRNA levels 200- to 700-fold higher than WT (Fig. 1a and Additional file 1: Raw data of Fig. 1a). *AtCYO1ox* lines grew similarly to WT, without any apparent defects in the younger vegetative stage. Two photosynthetic proteins, the large subunit of Rubisco (RBC-L) and LHCP, were expressed to the same degree as in WT (Fig. 1b). Plants harboring empty vector had gene expression levels comparable to WT. The mutant line *AtCYO1ox-4* showed the highest *AtCYO1* expression and was investigated further. Immunoblotting revealed AtCYO1 in the rosette leaves of line *AtCYO1ox-4* but not of WT (Fig. 1c). To determine the protein’s subcellular localization, chloroplasts were fractionated and immunoblotted (Fig. 1d). AtCYO1 was detectable in both the chloroplast pellet fraction containing the thylakoid LHCP as well as the stroma (supernatant) containing RBC-L. This distribution is distinct from that of native AtCYO1, which localizes only to thylakoids [5, 6].

### AtCYO1 overexpression causes leaves to stay green with functional PSII

**Fig. 2:**
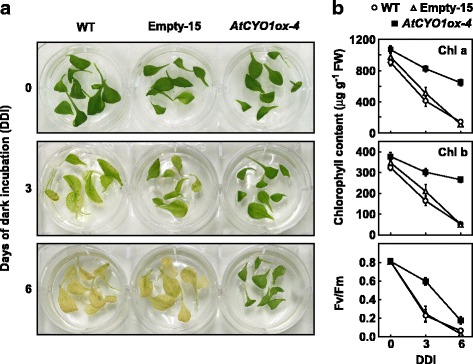
Stay-green phenotype of rosette leaves during dark incubation. **a** Leaves after 0, 3 and 6 days of dark incubation (DDI). **b** Changes in chlorophyll (Chl *a* and *b*) content and Fv/Fm. Data are expressed as the mean ± SE (*n* = 8-16)

We next investigated how AtCYO1 in leaves affects leaf senescence. Dark incubation of whole plants or leaf sections is a common technique for inducing senescence [17] and was applied here. Remarkably, *AtCYO1ox-4* stayed greener than WT following up to 6 days of dark incubation (Fig. 2a). Moreover, chlorophyll *a* and chlorophyll *b* content decreased more slowly in *AtCYO1ox-4* plants than in WT (Fig. 2b and Additional file 2: Raw data of Fig. 2b). Notably, some mutants with an impaired chlorophyll catabolic pathway commonly have stay-green phenotypes, as breakdown of chloroplast components proceeds normally during senescence while chlorophyll remains intact [17]. This type of stay-green is termed ‘cosmetic’ because the plants lose their photosynthetic activities, similar to the corresponding WT. To test whether the AtCYO1 overexpressed in leaves affected chlorophyll degradation alone, chlorophyll fluorescence (Fv/Fm) was monitored. A higher proportion of functional PSII (Fv/Fm) was observed in *AtCYO1ox-4* plants (Fig. 2b), suggesting that *AtCYO1* overexpression resulted in the maintenance of, at least, PSII complexes. The same experiments conducted using normal-growth light conditions revealed almost no decrease in chlorophyll content or Fv/Fm throughout the experiment in either *AtCYO1ox-4* or WT (Additional files 3 and 4: Figure S1 and Raw data of Figure S1). Thus, the senescence was induced primarily by darkness but not detachment or enclosure of the leaves. Stay-green phenotype was also observed in cotyledons (Additional file 5: Figure S2).

### Stay-green phenotype mediated by AtCYO1 overexpression is coordinated with decreased proteolysis in chloroplasts

**Fig. 3:**
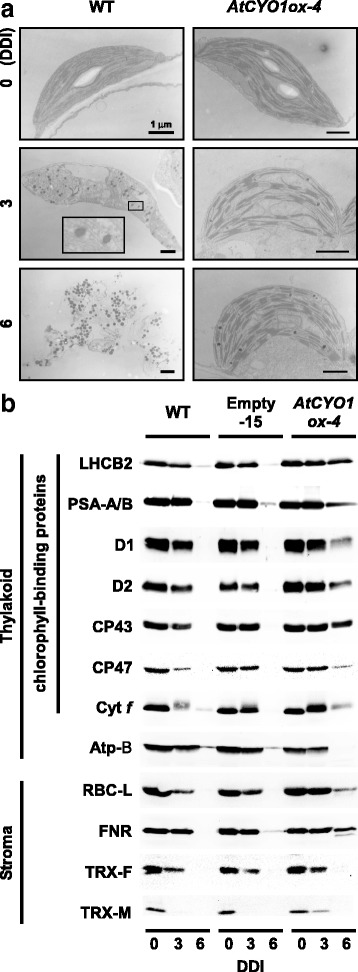
Effects of AtCYO1 on chloroplast stability following dark incubation. **a** Ultrastructure of chloroplasts from WT and *AtCYO1ox-4* leaves. Inset shows a 18X magnification of plastoglobules. **b** Immunoblotting of photosynthesis proteins in the thylakoid and stroma. LHCB2, D1, D2, CP43 and CP47 are proteins of PSII, and PSA-A and -B are proteins of PSI. Cyt *f*, subunit of the cytochrome *b*_6_*f* complex; Atp-B, subunit of ATPase; RBC-L, large subunit of Rubisco; FNR, ferredoxin-NADP^+^ reductase; TRX-F and -M, thioredoxins *f* and *m*. DDI, days of dark incubation

Following the chlorophyll fluorescence analysis, we expected the ultrastructure of chloroplasts to be more stable in *AtCYO1ox-4* because a rigid PSII structure (i.e., functional PSII) is required for the formation of stacked thylakoids (grana) [3]. Prior to dark incubation, no obvious difference in chloroplast ultrastructure was observed between *AtCYO1ox-4* and WT (Fig. 3a). Following dark incubation, however, plastoglobules (lipoprotein particles formed on thylakoids) increased in WT plants as the thylakoid membrane was breaking down. Many fewer plastoglobules appeared in *AtCYO1ox-4*, and the thylakoid remained intact at 6 DDI. Even at 10 DDI, when thylakoid membranes and chloroplast envelopes were no longer identifiable in WT, both grana and stroma thylakoids within the swollen envelope were still seen in *AtCYO1ox-4* (Additional file 6: Figure S3). The blistering of plastoglobules from the outer leaflet of the thylakoid membrane is essential for thylakoid disintegration [18]. Similar to *AtCYO1ox-4*, cosmetic stay-green mutants maintain thylakoid structures for a longer period and have fewer plastoglobules than WT [3, 19]. Therefore, we concluded that chlorophyll degradation preceded plastoglobule differentiation, and that *AtCYO1* overexpression negatively impacted the former process.

We further investigated chloroplast integrity using immunoblotting. AtCYO1 interacts with CP43, CP47, and LHCB1 but not D1 and D2 in PSII [15, 20] and with PSA-A and PSA-B in PSI [20]. At 6 DDI, these chloroplast proteins were still detectable in *AtCYO1ox-4* but not in WT (Fig. 3b). In addition to these PSII and PSI proteins, a subunit of the cytochrome *b*_6_*f* complex in the thylakoid membrane (cytochrome *f*) and several soluble proteins in the stroma (RBC-L, FNR, TRX-M) were also preferably maintained in *AtCYO1ox-4*. The maintenance of RBC-L was also supported by Coomassie staining following SDS-PAGE (Additional file 7: Figure S4). Taken together, these results indicated that *AtCYO1* overexpression broadly affected the maintenance of photosynthetic proteins during senescence.

### AtCYO1 overexpression affects chlorophyll degradation without altering gene expression

**Fig. 4:**
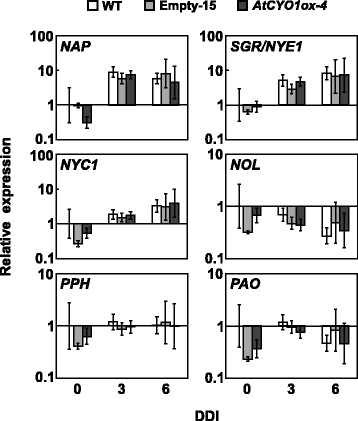
Expression of genes associated with chlorophyll degradation following dark incubation. Note the difference in scale of the *y* axis for each gene. Data are relative to WT at 0 days of dark incubation (DDI) and are expressed as the mean ± SE (*n* = 3)

Chlorophyll breakdown is a multistep reaction that requires chlorophyll catabolic enzymes (CCEs). The process is tightly regulated through gene expression levels [21]. Like cosmetic stay-green mutants, chlorophyll can remain if any of the catabolic reactions is suppressed. To investigate whether this was the case for *AtCYO1ox-4*, we first analyzed expression of six senescence-inducible genes involved in chlorophyll degradation. Among them, *NAP*, *SGR1* and *NYC1* were significantly upregulated in the dark, whereas *NOL* was downregulated (Fig. 4 and Additional file 8: Raw data of Fig. 4). Pheophytinase and pheophorbide *a* (Pheide *a*) oxygenase (PAO) were relatively unaffected, ranging within 0.2–1.3 fold of pre-dark levels in WT. Expression of these genes, including *SGR1*, was generally identical between *AtCYO1ox-4* and WT.

**Fig. 5:**
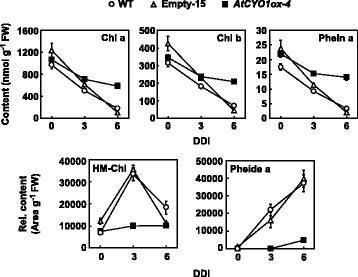
HPLC analysis of chlorophyll metabolites in leaves following dark incubation. Data are expressed as the mean ± SE (*n* = 5-6). For 7-hydroxymethyl chlorophyll *a* (HM-Chl) and pheophorbide *a* (Pheide *a*), relative content uses peak area of the chromatogram. DDI, days of dark incubation

Next, we investigated perturbations in chlorophyll intermediate catabolites. Pheophytin *a* (Phein *a*), the immediate catabolite of chlorophyll *a* [2], decreased closely in parallel with the chlorophylls (Fig. 5 and Additional file 9: Raw data of Fig. 5). Because Phein *a* is also a component of D1 [22], this correlation should represent the breakdown of PSII. To be decomposed, chlorophyll *b* needs to be initially converted to chlorophyll *a* through a two-step reduction catalyzed by CBR (NYC1 and NOL) and 7-hydroxymethyl chlorophyll *a* (HM-Chl) reductase (HCAR) [4, 23]. In WT leaves, HM-Chl increased at 3 DDI and then decreased at 6 DDI (Fig. 5), perhaps because most of the chlorophyll *b* was already depleted. In *AtCYO1ox-4*, on the other hand, HM-Chl increased only slightly during senescence. In the CCE cascade, Phein *a* is converted to Pheide *a* via the removal of phytol by pheophytinase, and this is converted to red chlorophyll catabolite (RCC) by PAO [24, 25]. During dark incubation, Pheide *a* accumulated continuously in WT but was only slightly elevated in *AtCYO1ox-4* (Fig. 5).

## Discussion

An increasing number of studies have investigated the importance and potential roles of CYO1/SCO2 in the assembly and/or maintenance of the thylakoid photosystem [5, 6, 8, 9, 14, 20]. However, self-destructive chloroplasts in defective mutants have hampered in vivo functional analyses because changes in chlorophyll biosynthesis, assembly of the photosystems, and resultant photosynthetic performance can be consequences of numerous defects in chloroplast biogenesis associated with an albino/pale-green phenotype [7]. Here, we genetically modified *Arabidopsis* to overexpress *AtCYO1* in leaves, where little or no AtCYO1 is otherwise expressed, providing a unique opportunity for functional analysis. Under dark-induced senescence, AtCYO1 overproduction affected the rate of chlorophyll degradation, slowed disassembly of PSI and PSII and maintained thylakoid integrity. The observed stabilization was not limited to chlorophyll protein complexes but was also seen for soluble proteins in the stroma. Although it is possible that ectopically overexpressed AtCYO1 may not behave like native AtCYO1, these results implicate redox regulation of protein disulfide bonds in the maintenance of chloroplast function.

During leaf senescence, CBR reduces chlorophyll *b* and SGR dechelates Mg^2+^ of chlorophyll *a*. Both are initial and rate-limiting steps for chlorophyll degradation [2] and can directly target chlorophyll-protein complexes [2, 4]. After removal of Mg^2+^, the other CCEs complete the chlorophyll degradation process to yield primary fluorescence chlorophyll catabolites in chloroplasts, and these catabolites are transported to vacuoles for further degradation [26]. The overexpression of *AtCYO1* delayed chlorophyll degradation without altering the expression of genes encoding CCEs in chloroplasts, suggesting that *AtCYO1* overexpression affected the catabolic reactions. There was no increased accumulation of catabolites of chlorophyll *a* and chlorophyll *b* in *AtCYO1ox-4* (Fig. 5), indicating that downstream catabolism was not inhibited. This was supported by the lack of photobleaching in *AtCYO1ox* leaves, which may have been the case when the reactions catalyzed by PAO or RCC reductase are impaired [24, 27]. When CBR is suppressed in either the *nyc1* or *nol* mutant, chlorophyll *a* is degraded to the same extent as in WT, whereas chlorophyll *b* is retained under dark incubation [3, 4]. On the other hand, suppression of SGR leads to retention of chlorophylls *a* and *b* owing to the maintenance of LHCP (i.e., the major sink for chlorophyll *b*), which is stabilized when chlorophyll *a* is bound to it [2, 19]. Accordingly, *AtCYO1* overexpression should have a negative impact on the primary step for chlorophyll degradation. It is now appreciated that chlorophyll degradation initiates disassembly of the PSI and PSII and that the dissociated apoproteins are degraded by proteases [2]. Like the SGR-defective mutants [2, 19], the intact chlorophylls in *AtCYO1ox* leaves stabilized both the core complexes and peripheral LHC (Fig. 3b). These chlorophyll-protein complexes, particularly LHCB [3], enabled the thylakoid structure to be maintained (Fig. 3a). Importantly, soluble proteins in the stroma, such as Rubisco, were preserved in *AtCYO1ox* leaves (Fig. 3b and Additional file 7: Figure S4). This is not the case for defective mutants of SGR or CBR, in which Rubisco degradation and chlorophyll catabolism are fully uncoupled so that Rubisco decays as quickly as in WT [17]. In contrast, tobacco leaves with suppressed CND41, the chloroplast-localized Rubisco protease, stay greener and have higher levels of Rubisco than WT during natural (non-induced) senescence [28]. Although the mechanism behind the coordinated breakdown in stromal proteins and the thylakoid photosystem has yet to be elucidated, the remaining Rubisco as well as other stromal proteins may impede the progress of senescence.

### Mechanisms of action of AtCYO1 under dark-induced senescence

There are two likely mechanisms for how *AtCYO1* overexpression slowed chloroplast degradation. First, AtCYO1 may interact with chlorophyll-protein complexes targeted by SGR. Protein disulfide reductase generally has low specificity in redoxin species [29], favoring a broad spectrum of AtCYO1 action. Many subunits of PSI (PSA-A and PSA-B) and PSII (D1, D2, CP43 and CP47, but not Cyt *f*) have Cys residues (Additional file 10: Table S1). In the thylakoid membrane, AtCYO1 is localized near the PSI/LHCI and PSII/LHCII complexes [5], potentially targeting either core complexes [20] or peripheral LHCB [15]. AtCYO1 may directly prevent these PS complexes from reacting with SGR. In line with this hypothesis, AtCYO1 may counteract oxidation of thiols and/or disulfide bonds in those apoproteins that would otherwise facilitate chlorophyll degradation. It is well known that senescence or stress leads to strong oxidative conditions inside chloroplasts owing to diminished reducing power generated by the dismantled photosynthetic electron transport chain [29]. Continuous darkness should lead to a similar change in the redox state because the photochemical reaction is shutdown. Furthermore, such progressive oxidation stimulates catabolism of Rubisco [30]. In *Arabidopsis*, the Rubisco complex consists of eight large subunits with nine conserved Cys residues plus eight small subunits with five conserved Cys residues (Additional file 10: Table S1). A shift in the redox status of these Cys residues triggers conformational changes in the Rubisco complex, causing effects ranging from reversible inactivation to proteolytic sensitization towards oxidation [30]. The protein disulfide reductase/isomerase assays [5] implied the hydrophilic active site for the AtCYO1 expressed in *E. coli*. Indeed, AtCYO1 is rich in hydrophilic amino acid groups (Additional file 11: Figure S5). Moreover, in *AtCYO1ox* leaves, AtCYO1 appeared to localize to the stroma as well as the thylakoid fractions (Fig. 1d). Therefore, we speculate that *AtCYO1* overexpression is involved in the chemical reduction of Rubisco, delaying its oxidation in the pre-proteolytic phase. Notably, most of the CCEs, including SGR1, also have conserved Cys residues (Additional file 10: Table S1). Further studies are needed to clarify in vivo targets of AtCYO1. It is well known that carbon metabolism enzymes are activated by thioredoxins upon illumination. Electrons from the PSI are initially received by ferredoxin and ultimately delivered to NADPH in the Calvin cycle. In parallel, a smaller proportion of electrons is likely directed to the thioredoxins, which in turn reduce the disulfide bonds of carbon metabolism enzymes [31]. For AtCYO1, reduced glutathione but not NADPH appears to be the in vivo reducer [20]. Reduced glutathione and its oxidized form are very abundant in the stroma (up to the millimolar range), and the ratio of reduced/oxidized glutathione is high under both light and darkness, creating a redox buffer pool [29]. Unlike in the ferredoxin-thioredoxin system, AtCYO1 may be active in the glutaredoxin system in darkness.

In the second mechanism, AtCYO1 may be involved in the (dis)assembly of PSII supramolecular complexes. Lu et al. [15] suggested that both AtCYO1 and THF1/NYC4 function in chloroplast vesicle transport during chloroplast biogenesis. In the *thf1* knockout mutant, chloroplasts in variegated leaf sectors accumulate vesicles instead of normal thylakoids [11, 12]. Likewise, normally shaped chloroplasts from *atsco2* cotyledons accumulate vesicles [14]. Both AtCYO1 and THF1 interact with LHCB proteins, suggesting that LHCBs are transported to thylakoid membranes within these vesicles [13, 14]. The loss of function in the *thf1/nyc4* mutant resulted in stay-green phenotypes with enhanced stability of functional PSII in both *Arabidopsis* [13] and rice [16]. Huang et al. [13] found that the largest PSII megacomplex remained at higher levels in the *thf1* mutant than in WT. Because SGR can react with protein-bound chlorophyll *a* at the thylakoid [2], SGR may be unable to target the megacomplexes. Whereas THF1 negatively affects accumulation of megacomplexes, AtCYO1 may positively affect such accumulation, resulting in maintenance of D1 and D2 (Fig. 3) despite the lack of direct interactions [20]. This view is supported by recent findings that the formation of super- and megacomplexes was especially impaired in the *atcyo1* mutant [9]. What was striking in that study was that the impairment occurred in leaves as well as cotyledons under short-day conditions (8 h light/16 h dark). In the absence of AtCYO1, THF1 could readily decouple megacomplexes to yield smaller complexes. Destruction of the chloroplasts may then proceed, mediated by either SGR during a dark period or reactive oxygen species generated by the disproportional photosystem during a light period. However, formation or retention of megacomplexes does not necessarily explain the retention of Rubisco in our present study. Indeed, the *thf1/nyc4* mutant sustains thylakoid photosystems but not Rubisco during senescence [13, 16].

## Conclusions

Overall, either or both of these proposed modes of action could contribute to delayed senescence in the *AtCYO1* overexpression mutant. Moreover, whatever the involvement of AtCYO1, redox in the chloroplast must be the key regulator of protein disulfide reductase/protein disulfide isomerase activities. We conclude that redox modulation of Cys residues and/or disulfide bonds is one of the critical factors regulating leaf senescence and/or acclimation of photosynthesis for plants grown under particular dark/light regimes.

## Methods

### Plant growth

All *Arabidopsis thaliana* wild-type and transgenic lines were Columbia-0 ecotype. Plnats were grown on 1/2 Murashige and Skoog medium plates without sucrose at 23°C under fluorescent light (60–70 μmol m^− 2^ s^− 1^) under long-day conditions (16 h light/8 h dark). For dark/light experiments, green rosette leaves were detached from 3-week-old plants and placed on the same growth medium for up to 6 days in continuous darkness or long-day (control) conditions at 23°C. Sixteen leaves (two leaves from a plant) were used for a single experiment and were subsequently sampled under dim light (< 5 μmol m^− 2^ s^− 1^ PAR). Whole plants grown on the same growth medium for 2 weeks were subjected to the same dark incubation for up to 10 days. Shoots from 30 plants were sampled under dim light before and after the dark incubation.

### Construction of the *AtCYO1* overexpression vector and lines

The *AtCYO1* cDNA fragment was amplified using primers containing sites for *Xba*I (5′ GGGTCTAGATTCTCGTCTCAATGTTCCGATTATACCCTA-3′) and *Bam*HI (5′-GGGGGATCCAACGAACCCAAGCTTACATGCAAAAATGGG-3′). Amplified DNA fragments were digested with *Xba*I and *Bam*HI and ligated into the binary vector pBI121GS [32]. The sequence of the DNA fragment was confirmed, and the vector was introduced into *Arabidopsis* (Col-0) via *Agrobacterium tumefaciens* (strain GV3101) using the floral-dip method [33].

### Quantitative reverse transcription-PCR

The NucleoSpin RNA kit (Takara, Japan) and ReverTra Ace qPCR RT Master Mix (Toyobo, Japan) were used for preparation of total RNA and cDNA, respectively. The transcript level was determined using the ABI Prism 7300 sequence detection system (Thermo Fisher Scientific, US) using the SYBR Fast qPCR kit (Kapa Biosystems, US). The primers used for amplification are listed (Additional file 12: Table S2).

### Pigment analysis

Total chlorophyll was extracted from N_2_-ground rosette leaves using ice-cold 80% acetone at 4 °C, and chlorophyll *a* and *b* content was quantified spectrophotometrically as described by Porra et al. [34]. Chlorophyll metabolites were measured by HPLC using a Symmetry C8 column (Waters, US) and a photodiode-array detector (SPD-M10A; Shimadzu, Japan) according to Zapata et al. [35] and Tanaka et al. [24]. For HPLC analysis, extraction was performed in 100% acetone at − 30°C.

### Chlorophyll fluorescence measurement

Maximal photochemical efficiency of PSII (Fv/Fm) was measured using a JUNIOR-PAM fluorometer (Walz, Germany) according to the manufacturer’s instructions.

### Transmission electron microscopy

Leaves were fixed overnight in 4% paraformaldehyde and 1% glutaraldehyde and then postfixed with 1% osmium tetroxide in 50 mM cacodylate buffer for 2 h at room temperature. The samples were dehydrated with an ethanol series (50 to 100%), which was then replaced with propylene oxide. Samples were infiltrated overnight with a 1:1 (*v*/v) solution of propylene oxide and Epon 812 resin (TAAB Laboratories Equipment, UK). The samples were subsequently embedded in Epon resin, which was allowed to polymerize at 60 °C for 72 h. Ultrathin sections were cut using an ultramicrotome (Ultracut-UCT; Leica,Germany) and mounted on a nickel grid. The sections were stained with 4% uranyl acetate and lead citrate and observed using a transmission electron microscope (H-7600; Hitachi, Japan).

### SDS-PAGE and immunoblotting

Leaves (100 mg fresh weight) were homogenized in 400 μl extraction buffer (15 mM Tris-HCl, pH 8.0, 50 mM NaCl, 0.1 mM EDTA, 1% SDS), incubated for 5 min on ice, and then centrifuged (10,000 × *g*, 5 min, 4°C). The supernatant was used for immunoblotting. All antibodies used are described elsewhere [5, 8]. RBC-L and LHCP were visualized by staining SDS-PAGE gel with Coomassie Brilliant Blue R250.

## Additional files

Additional file 1: Raw data of Fig. 1a. (XLSX 20 kb)

Additional file 2: Raw data of Fig. 2b. (XLSX 41 kb)

Additional file 3: **Figure S1.** Same experiment as in Fig. 2, except that samples were incubated under normal growth light conditions (mean ± SE; *n* = 16). (PDF 9171 kb)

Additional file 4: Raw data of **Figure S1**. (XLSX 37 kb)

Additional file 5: **Figure S2.** Cotyledons at 9 DDI. (PDF 77 kb)

Additional file 6: **Figure S3.** Ultrastructure of chloroplasts at 10 DDI. (PDF 3868 kb)

Additional file 7: **Figure S4.** Maintenance of RBC-L and LHCP at 10 DDI as indicated by Coomassie staining following SDS-PAGE. (PDF 586 kb)

Additional file 8: Raw data of Fig. 4. (XLSX 74 kb)

Additional file 9: Raw data of Fig. 5. (XLSX 56 kb)

Additional file 10: **Table S1.** Number of Cys residues in chloroplast proteins in *Arabidopsis*. (PDF 73 kb)

Additional file 11: **Figure S5.** Hydropathic character of AtCYO1, with RBC-L for comparison. Amino acid sequences were analyzed using the Kyte-Doolittle hydropathy plot (http://gcat.davidson.edu/DGPB/kd/kyte-doolittle.htm). Positive and negative values indicate hydrophobic and hydrophilic residues, respectively. (PDF 210 kb)

Additional file 12: **Table S2.** Primers for quantitative reverse transcription-PCR. (PDF 70 kb)

## Abbreviations

CBR: Chlorophyll b reductase
CCE: Chlorophyll catabolic enzyme
Cys: Cysteine
DDI: Days of dark incubation
HCAR: HM-Chl reductase
HM-Chl: 7-hydroxymethyl chlorophyll *a*
LHC: Light-harvesting complex
LHCB: LHCP in photosystem II
LHCP: LHC protein
PAO: Pheide a oxygenase
Pheide a: Pheophorbide a
Phein a: Pheophytin a
PS: Photosystem
RBC-L: Rubisco large subunit
SGR: STAY-GREEN
