# Supplementary material for: Overexpression of the protein disulfide isomerase AtCYO1 in chloroplasts slows dark-induced senescence in Arabidopsis
Source: BMC Plant Biol. 2018 May 4;18:80. doi: 10.1186/s12870-018-1294-5 (PMC5935949; doi:10.1186/s12870-018-1294-5)
Supplement: Supplementary file 3 — Figure S1. Same experiment as in Fig. 2, except that samples were incubated under normal growth light conditions (mean ± SE; n = 16). (PDF 9171 kb) [file 12870_2018_1294_MOESM3_ESM.pdf]

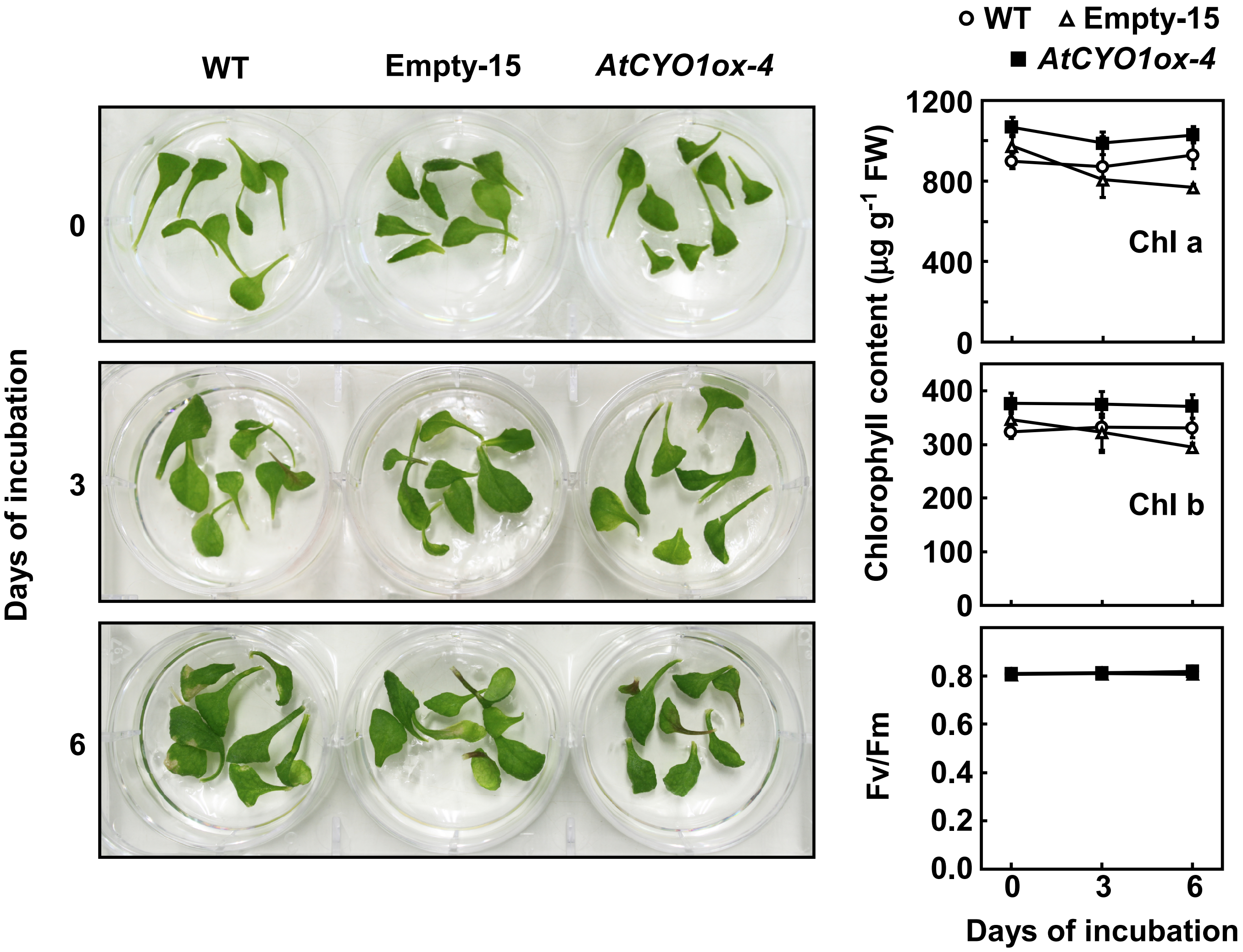

Figure S1 Same experiment as in Figure 2, except that samples were incubated under normal growth light conditions (mean ± SE; *n* = 16).
