# Supplementary figures and images for: Overexpression of the protein disulfide isomerase AtCYO1 in chloroplasts slows dark-induced senescence in Arabidopsis
Source: BMC Plant Biol. 2018 May 4;18:80. doi: 10.1186/s12870-018-1294-5 (PMC5935949; doi:10.1186/s12870-018-1294-5)

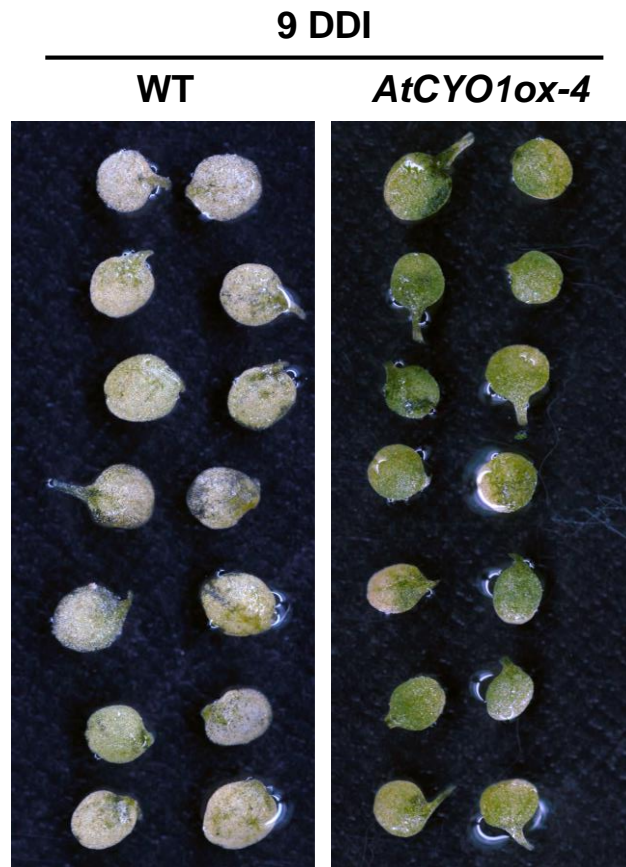

**Figure S2** Cotyledons at 9 DDI.

Whole plants were subjected to the dark incubation.

Supplement: Supplementary file 5 — Figure S2. Cotyledons at 9 DDI. (PDF 77 kb) [file 12870_2018_1294_MOESM5_ESM.pdf]

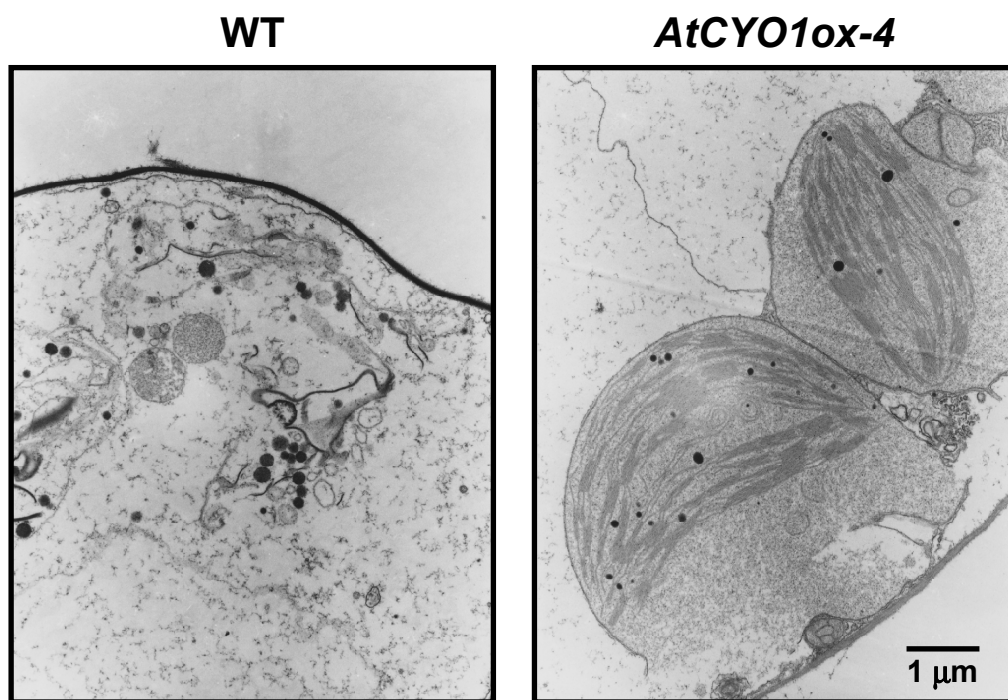

**Figure S3** Ultrastructure of chloroplasts at 10 DDI.

Supplement: Supplementary file 6 — Figure S3. Ultrastructure of chloroplasts at 10 DDI. (PDF 3868 kb) [file 12870_2018_1294_MOESM6_ESM.pdf]
