# Supplementary material for: Overexpression of the protein disulfide isomerase AtCYO1 in chloroplasts slows dark-induced senescence in Arabidopsis
Source: BMC Plant Biol. 2018 May 4;18:80. doi: 10.1186/s12870-018-1294-5 (PMC5935949; doi:10.1186/s12870-018-1294-5)
Supplement: Supplementary file 7 — Figure S4. Maintenance of RBC-L and LHCP at 10 DDI as indicated by Coomassie staining following SDS-PAGE. (PDF 586 kb) [file 12870_2018_1294_MOESM7_ESM.pdf]

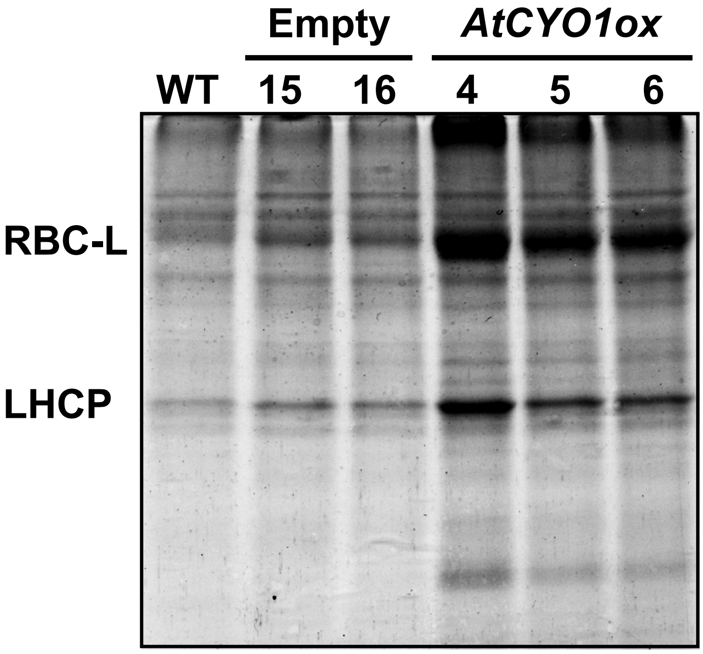

Figure S4 Maintenance of RBC-L and LHCP at 10 DDI as indicated by Coomassie staining following SDS-PAGE.
