# Supplementary material for: Overexpression of the protein disulfide isomerase AtCYO1 in chloroplasts slows dark-induced senescence in Arabidopsis
Source: BMC Plant Biol. 2018 May 4;18:80. doi: 10.1186/s12870-018-1294-5 (PMC5935949; doi:10.1186/s12870-018-1294-5)
Supplement: Supplementary file 10 — Table S1. Number of Cys residues in chloroplast proteins in Arabidopsis. (PDF 73 kb) [file 12870_2018_1294_MOESM10_ESM.pdf]

Table S1 Number of Cys residues in chloroplast proteins in *Arabidopsis.*

Protein	Number of Cys residues
PSI	
PSA-A	4
PSA-B	2
PSA-K	2
PSA-C	9
PSA-D	1
PSA-F	3
PSA-N	5
LHCA1	3
LHCA2	2
LHCA3	0
LHCA4	1
PSII	
D1	2
D2	4
CP47	3
CP43	4
LHCB1	1
LHCB2	1
LHCB3	1
Rubisco	
RBC-L	9
RBC-S	5
CCEs	
NYC1	8
NOL	5
HCAR	10
SGR1	8
SGR2	9
SGRL	6
PPH	6
PAO	10
RCCR	4
