# Supplementary material for: Overexpression of the protein disulfide isomerase AtCYO1 in chloroplasts slows dark-induced senescence in Arabidopsis
Source: BMC Plant Biol. 2018 May 4;18:80. doi: 10.1186/s12870-018-1294-5 (PMC5935949; doi:10.1186/s12870-018-1294-5)
Supplement: Supplementary file 11 — Figure S5. Hydropathic character of AtCYO1, with RBC-L for comparison. Amino acid sequences were analyzed using the Kyte-Doolittle hydropathy plot (http://gcat.davidson.edu/DGPB/kd/kyte-doolittle.htm). Positive and negative values indicate hydrophobic and hydrophilic residues, respectively. (PDF 210 kb) [file 12870_2018_1294_MOESM11_ESM.pdf]

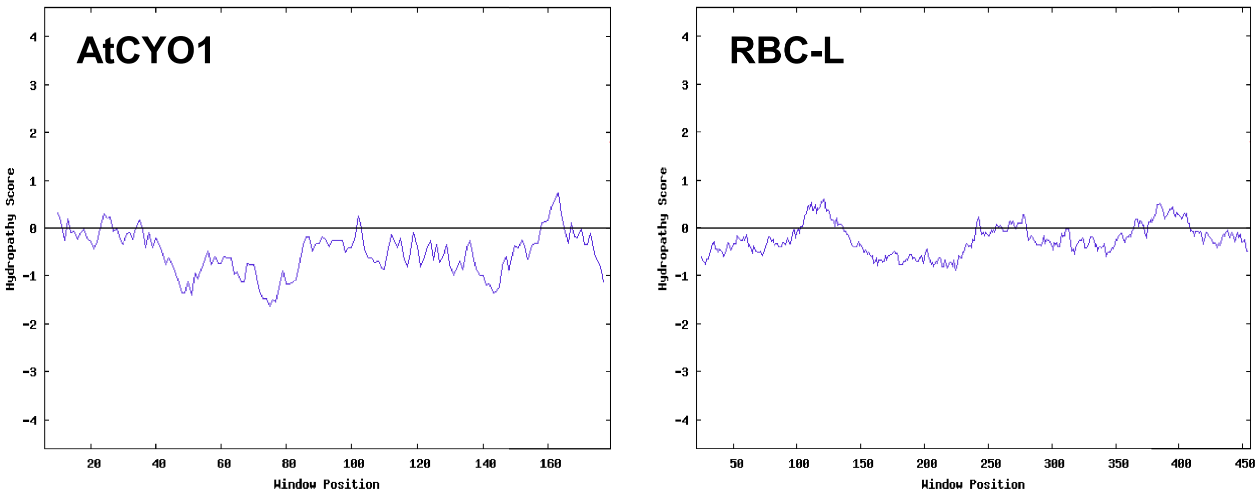

Figure S5 Hydropathic character of AtCYO1, with RBC-L for comparison. Amino acid sequences were analyzed using the Kyte-Doolittle hydropathy plot (http://gcat.davidson.edu/DGPB/kd/kyte-doolittle.htm). Positive and negative values indicate hydrophobic and hydrophilic residues, respectively.
