# Supplementary material for: Overexpression of the protein disulfide isomerase AtCYO1 in chloroplasts slows dark-induced senescence in Arabidopsis
Source: BMC Plant Biol. 2018 May 4;18:80. doi: 10.1186/s12870-018-1294-5 (PMC5935949; doi:10.1186/s12870-018-1294-5)
Supplement: Supplementary file 12 — Table S2. Primers for quantitative reverse transcription-PCR. (PDF 70 kb) [file 12870_2018_1294_MOESM12_ESM.pdf]

Table S2 Primers for quantitative reverse transcription-PCR.

Gene	Sequence	
	Forward	Reverse
Reference		
UBQ10	CACACTCCACTTGGTCTTGCGT	TGGTCTTTCCGGTGAGAGTCTTCA
Target		
NAP	CGAAGCAGAGAGAAGAACTGAA	CAAATGAGCCAGCGAACAC
SGR1	GGGAAAATGTCGCTTCACG	AGCCTTCAACACCACAGGTAG
NYC1	GCAGAGAACAGGACGAGGTT	CGCAAACAACAGAAAGAGAGAA
NOL	CCGACTTACATCCGTTTCCTAA	CTGTTCTTCCTTGCTCCCAAC
PPH	AATCCCCAACGCTCCATACT	CTTCAAAACCACCAGACTCCA
PAO	CCCAGGCAGACCGTTTTGT	TGACTCTTACCATGCCGTCTGA
